# To Preserve or Not To Preserve: A Prospective Cohort Study on the Role of the Cervix in Post-Hysterectomy Sexual Functioning

**DOI:** 10.7759/cureus.68876

**Published:** 2024-09-07

**Authors:** Mohamed Ferhi, Nadia Marwen, Ameni Abdeljabbar, Jihenne Mannai

**Affiliations:** 1 Psychiatry, Ibn El Jazzar University Hospital, Kairouan, TUN; 2 Obstetrics and Gynecology, Ibn El Jazzar University Hospital, Kairouan, TUN

**Keywords:** asex, female sexual health, female sexuality, fsfi, hysterectomy, orgasm, prospective cohort, psychiatric effects, sexual desire, total hysterectomy

## Abstract

Background

Hysterectomy remains the most commonly performed gynecologic procedure worldwide, undertaken primarily for benign pathologies. The choice between total hysterectomy (TH) and subtotal hysterectomy (STH) has been debated, particularly with respect to its impact on sexual functioning (SF).

Objective

This study aimed to assess the impact of TH versus STH on SF and to determine whether preservation of the cervix in STH offers advantages in terms of postoperative SF.

Methods

A prospective cohort study was conducted at Ibn El Jazzar University Hospital, Kairouan, Tunisia, involving women aged 40 to 65 years who underwent hysterectomy for benign conditions between January 2, 2020, and December 31, 2021. SF was evaluated using the Arizona Sexual Experiences Scale (ASEX) and the Female Sexual Function Index (FSFI) before and six months after surgery. Statistical analyses were performed using SPSS version 26.

Results

Sixty women were included, with 30 undergoing TH and 30 undergoing STH. Postoperative evaluations revealed improvements in SF in both groups without statistically significant differences between TH and STH in terms of SF scores or the timeline for resuming sexual activity.

Conclusions

Hysterectomy, regardless of the technique used, appears to have a positive impact on SF, largely attributed to symptomatic relief. Therefore, the choice between TH and STH should consider factors beyond potential differences in SF outcomes. Women considering hysterectomy for benign indications should be informed of these findings to aid in the decision-making process regarding their surgical options.

## Introduction

Hysterectomy, the surgical removal of the uterus, is the most frequently performed gynecological procedure worldwide [1]. Hysterectomies are classified into two types: those performed for carcinological diseases and those performed for benign pathologies, which account for the majority of cases. The benign conditions that warrant hysterectomy include uterine fibroids, persistent abnormal uterine bleeding that does not respond to medical treatment, endometriosis, adenomyosis, and uterine prolapse [2]. Total hysterectomy (TH) encompasses the surgical excision of both the uterus and the cervix, whereas subtotal hysterectomy (STH) is characterized by the excision of the uterus while preserving the cervical stump.

Although the distinction between TH and STH is crucial for surgical planning, it is also imperative to consider the broader spectrum of potential complications associated with hysterectomy. Hemorrhagic complications are the most common, occurring in approximately 4.7% of cases regardless of the surgical approach [3]. Other potential complications include vesicoureteral injuries, digestive complications, and static pelvic disorders [3]. A significant complication reported in the literature is the impact of hysterectomy on sexual functioning (SF) [4]. The controversy surrounding the role of the uterus and cervix in the female sexual response stems from differences in innervation and brain projections between the cervix and clitoris. Some authors argue that the cervix is involved in female orgasm. Lopès P and Poudat F-X, in the Manual of Sexology, proposed mechanisms explaining the potential involvement of the uterine cervix in the orgasmic response [5]. They suggested that the uterus likely plays a role in the female sexual response through the round ligaments, and mobilization of the cervix during penetration may stimulate the vulva by stretching these ligaments. Similarly, Komisaruk BR and Whipple B [6] proposed the existence of three distinct entities that contribute to female orgasms: cervical, vaginal, and clitoral orgasms. On the other hand, recent functional imaging studies have shed light on the crucial role of the brain in the orgasmic response [7]. This suggests that orgasm is not merely a reflexive reaction but rather a complex process centered on the brain. Consequently, the brain, rather than the cervix, vagina, or clitoris, can be considered the center of desire and pleasure [7]. Furthermore, any major pelvic surgery or injury has the potential to damage the nerves and blood vessels that supply the vagina and clitoris, which can affect SF after surgery. Despite these theoretical considerations, empirical studies directly comparing the sexual outcomes of TH versus STH are sparse and inconclusive [8-11].

Given the nuanced debate surrounding the physiological and psychological roles of the cervix in sexual satisfaction and response, the decision between TH and STH is of significant concern. This decision not only impacts the surgical management of benign gynecological conditions but also has potential consequences for postoperative SF. The preservation of the cervix in STH, as opposed to complete removal in TH, raises pertinent questions about their respective impacts on sexual health outcomes. Therefore, this study aimed to fill the literature gap by providing a comprehensive comparison of outcomes of SF in patients undergoing TH versus STH for benign conditions.

## Materials and methods

Study design and setting

This study was carried out in the Department of Obstetrics and Gynecology at Ibn El Jazzar University Hospital, Kairouan, Tunisia. Consecutive women between the ages of 40 and 65 years who had received a hysterectomy for a benign indication from January 2, 2020, to December 31, 2021, were selected for inclusion in this prospective cohort study.

Eligibility criteria

The exclusion criteria were defined to ensure minimized confounding factors that affect SF. Participants were excluded if they had preexisting psychiatric conditions, including major depressive disorder, anxiety disorders, bipolar disorder, schizophrenia, and sexual disorders diagnosed according to the Diagnostic and Statistical Manual of Mental Disorders, Fifth Edition (DSM-5) criteria. Verification of psychiatric conditions was carried out through communication with treating psychiatrists for patients under psychiatric care. Additionally, women were excluded if they had used, within the three months prior to the study, any medications known to significantly alter SF. This included selective serotonin reuptake inhibitors (SSRIs), tricyclic antidepressants, antipsychotics, antiandrogens, specific antihypertensives (e.g., beta-blockers), and hormone replacement therapy. Other exclusion criteria included the need for concomitant surgical interventions (e.g., prolapse repair), hysterectomies performed for hemostatic or carcinological reasons, and incidental discovery of malignancy in the hysterectomy sample postoperatively. Exclusion also applied to individuals who reported no sexual activity within six months prior to surgery, with sexual activity defined according to the WHO as encompassing any sexual acts undertaken for pleasure, whether with a partner or by masturbation. Further exclusion criteria included loss of a sexual partner during the study period, incomplete questionnaire responses, any medical condition that interferes with the understanding of the evaluation, and refusal to participate in the study.

Detailed surgical procedures and perioperative protocols

Upon admission to the study, a comprehensive initial assessment was performed for all participants to document their medical history and perform a thorough gynecological evaluation. This evaluation included a gynecologic examination, transvaginal ultrasound, and the collection of samples for basic laboratory tests. To standardize perioperative care and minimize variability in treatment outcomes, a uniform perioperative management protocol was implemented across all participating hospitals. This protocol included the administration of perioperative prophylaxis for deep vein thrombosis, using low molecular weight heparin (ENOXA 4000 UI ANTI-XA/0.4ML) administered subcutaneously. Analgesia was the same for all patients. Additionally, to reduce the risk of infection at the surgical site, a single dose of prophylactic antibiotic was administered intravenously during surgery.

The decision-making process regarding the surgical technique used for hysterectomy was primarily guided by the personal preference and technical expertise of the operating gynecologist. However, the indication for hysterectomy, determined by the specific medical condition of the patient, also influenced the choice of surgical technique. The surgical procedures for all types of hysterectomy were discussed with each patient, and the suitable hysterectomy type was selected after the patient's informed consent.

Data collection

Data were collected through direct interviews with patients. An initial evaluation interview was conducted the day before surgery, and a second interview was conducted 6 months after surgery during the outpatient visit. The same interviewer asked questions in a calm and private setting, respecting the patient’s privacy. Both groups were monitored in the same way throughout the study period. Medical and obstetric records were used to collect data on the sociodemographic and clinical characteristics of the patients. For the data related to the operation, we consulted the operating reports and the monitoring sheets.

Assessment measures

SF was assessed using two questionnaires. The first questionnaire was the Arizona Sexual Experiences Scale (ASEX). It is a simple and brief questionnaire used to identify possible sexual dysfunction. It consists of five items that assess sexual drive, sexual arousal, vaginal lubrication, orgasm, and satisfaction. Each item is rated on a scale of 1 to 6, ranging from hyperfunctioning to hypofunction. A total ASEX score greater than or equal to 18 is considered the cutoff point for sexual dysfunction. The ASEX scale has a high specificity of 95.52%, a sensitivity of 70%, a positive predictive value (PPV) of 89.66%, and a negative predictive value (NPV) of 85.33%. It is also well-accepted by patients, making it suitable for assessing changes in SF over time [12,13].

The second questionnaire was the Female Sexual Function Index (FSFI). The FSFI is a widely used measurement instrument in sexual medicine. It consists of 19 questions that assess six components: desire, sexual arousal, vaginal lubrication, orgasm, sexual satisfaction, and pain. Each item is scored on a scale from 0 to 5 or from 1 to 5. The score for each domain is obtained by multiplying the score by a corresponding factor for each element. The maximum score for all assessed domains is 6. A total score of 26.55 is the cutoff value for diagnosing sexual dysfunction. The FSFI has been extensively validated and used in research on sexual medicine [14,15].

Ethical considerations

The ethics committee of Ibn El Jazzar University Hospital gave official approval for the study to be carried out (under approval number 4523). The women had been informed of the study's goal, procedure, benefits, nature, follow-up, and right to withdraw at any time without explanation and had given their written consent. Through the coding of all the data and the protection of the acquired data, the confidentiality and anonymity of each woman was guaranteed.

Statistical analysis

Statistical analysis was performed using the SPSS, version 26 software. Qualitative variables were described using observed numbers (n) and frequencies (%), while quantitative variables were examined for distribution using skewness and kurtosis coefficients, along with normality tests such as the Shapiro-Wilk, Kolmogorov-Smirnov, and Anderson-Darling tests, depending on the sample size and distribution characteristics. Variables following a normal distribution were described using means and standard deviations, while medians and interquartile ranges were used for variables not normally distributed. The association between two categorical variables was assessed using the Chi-square test, with Fisher's exact test applied when Chi-square test assumptions were not met. For comparing a qualitative variable with a quantitative one, Student's t-test was used for normally distributed data, and the Mann-Whitney U test for two independent samples was employed for data that did not follow a normal distribution. A significance level (p-value) of 0.05 was established for all tests.

## Results

Sociodemographic and clinical characteristics

**Figure 1:**
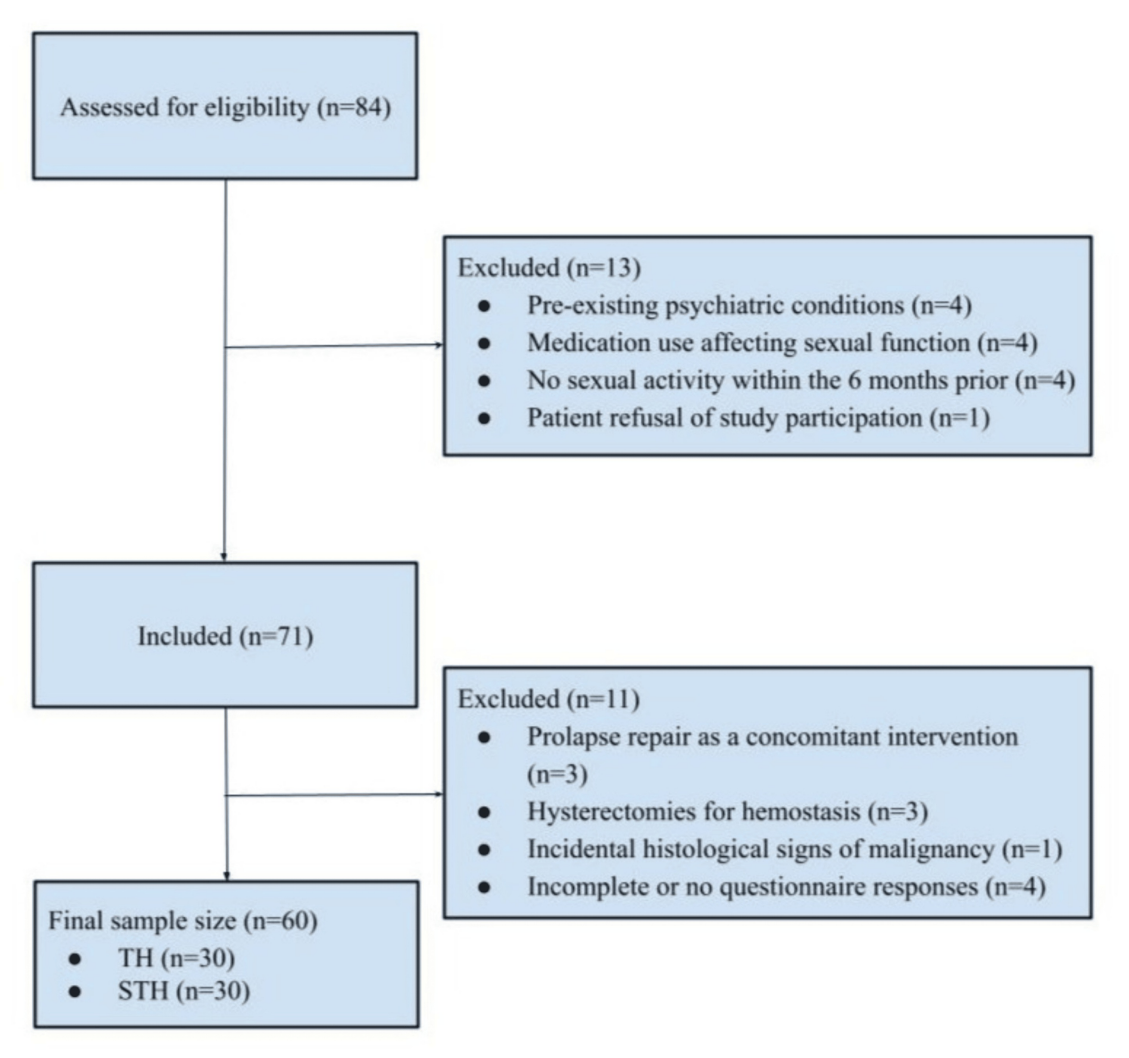
Flowchart of participant recruitment and selection. TH: Total hysterectomy; STH: Subtotal hysterectomy.

A total of 84 patients were evaluated for eligibility, and 60 patients were included, with 30 women in each group, as shown in Figure 1. Table 1 presents the sociodemographic characteristics of the patients. The average age was slightly over 52 years. The mean duration of marriage was around 23 years. When examining educational levels, a quarter of the participants had no formal education, slightly more than a quarter had completed primary education, another quarter had secondary education, and one-fifth had achieved higher education. Almost half of the group was classified as having a poor economic level. Lifestyle habits revealed a low prevalence of tobacco use (less than 10%) and regular physical activity (slightly more than 8%), and no participants reported alcohol consumption. BMI classification showed a small fraction (less than 2%) as underweight, more than half as having normal BMI, around one-fifth were overweight, and slightly less than a third were classified as obese.

**Table 1:** Sociodemographic characteristics of the study participants (n=60). TH: Total Hysterectomy; STH: Subtotal Hysterectomy; n: Frequencies; %: Percentages. Notes*:*^ a^ in years; ^b^ percentages are expressed out of the total number of participants.

Characteristics	Total, n (%)^ b^, n=60	TH, n(%), n=30	STH, n (%), n=30
Mean age(SD)^a^	52.1 (7.3)	55.87 (6.8)	48.3 (5.6)
Age intervals^a^			
(40-49)	24 (40.0)	-	-
(50-59)	22 (37.0)	-	-
(60-69)	14 (23.0)	-	-
Marriage duration ^a^			
Mean (range)	23.5 (15.0-32.0)	26.5 (19.7-32.0)	20 (15.0-23.2)
> 20	31 (52.0)	-	-
< 20	29 (48.0)	-	-
Educational level			
Illiterate	15 (25.0)	21 (70.0)	11 (36.7)
Primary	17 (28.0)		
Secondary	16 (27.0)	9 (30.0)	19 (63.3)
Superior	12 (20.0)		
Poor economic level	27 (45.0)	14 (46.7)	13 (43.3)
Lifestyle habits			
Tobacco use	4 (6.7)	1 (3.3)	3 (10.0)
Regular physical activity	5 (8.3)	1 (3.3)	4 (13.3)
Alcohol consumption	0 (0.0)	-	-
Body Mass Index			
Underweight	1 (1.7)	19 (63.3)	13 (43.3)
Normal	31 (51.7)		
Overweight	11 (18.3)	11 (36.7)	17 (56.7)
Obese	17 (28.3)		

Table 2 displays the clinical and operative characteristics of the patients. Diabetes was prevalent in approximately one-fourth of the participants. Median gravidity and parity were reported as four pregnancies and two live births, respectively. In particular, more than 90% of the cohort had a history of childbirth, with around 14.5% undergoing cesarean sections and a predominant majority experiencing vaginal deliveries. The onset of menopause occurred at a mean age of 46 years, affecting 70% of the participants.

**Table 2:** Clinical and operative characteristics of the study participants (n=60). TH: Total Hysterectomy; STH: Subtotal Hysterectomy; n: Frequencies; %: Percentages; IQR: Interquartile Range. Notes*:* ^a^ Percentages are expressed out of the total number of participants.

Characteristics	Total, n (%)^a ^ , n=60	HT, n (%), n=30	HT, n (%), n=30
History of diabetes	14 (23.3)	12 (40.0)	2 (6.7)
Gravidity, median (IQR)	4 (3-6)	6 (3-8)	3 (3-4)
Nulligravid	5 (8.3)	3 (10.0)	2 (6.7)
Parity, median (IQR)	2 (3-5)	5 (3-8)	3 (3-4)
Nulliparous	5 (8.3)	3 (10.0)	2 (6.7)
History of childbirth	55 (91.7)	-	-
History of a cesarean section	8 (14.5)	-	-
History of a vaginal delivery	47 (85.5)	-	-
Number of vaginal deliveries, median (IQR)	3 (2-6)	5 (3-8)	3 (0-3)
History of an instrumental extraction	3 (6.4)	1 (3.3)	2 (6.7)
Menopause	42 (70.0)	25 (83.3)	17 (56.7)
Menopause age of onset, mean (range)	46 (39-55)	-	-
Preoperative clinical symptoms			
Chronic pelvic pain	30 (50.0)	10 (33.3)	20 (66.7)
Abnormal uterine bleeding	18 (30.0)	8 (26.7)	10 (33.3)
Sensation of a ball in the vagina	12 (20.0)	12 (40.0)	0 (0.0)
Surgical indications			
Uterine leiomyomas	26 (43.3)	10 (33.3)	16 (53.3)
Urogenital prolapses	12 (20.0)	12 (40.0)	0 (0.0)
Adenomyosis	12 (20.0)	3 (10.0)	9 (30.0)
Abnormal uterine bleeding	10 (16.7)	5 (16.7)	5 (16.7)
Intervention			
Total hysterectomy	30 (50.0)	-	-
Subtotal hysterectomy	30 (50.0)	-	-
Surgical approach			
Vaginal route	14 (23.3)	14 (46.7)	0 (0.0)
Laparotomy	46 (76.6)	16 (53.3)	30 (100.0)
Bilateral adnexectomy	43 (71.7)	25 (83.3)	18 (60.0)
Post-operative complications			
Bladder wounds	3 (5.0)	-	-
Postoperative peritonitis	1 (1.7)	-	-
Surgery site infection	2 (3.4)	-	-

Half of the participants reported chronic pelvic pain, while one-third and one-fifth of the study group noted abnormal uterine bleeding and sensation of a ball in the vagina, respectively. The surgical intervention predominantly involved laparotomy, chosen in 76.6% of cases. Postoperative complications were rare (10.0%), with bladder wounds, postoperative peritonitis, and surgical site infections observed in a minority of cases.

Sexual functioning results

**Figure 2:**
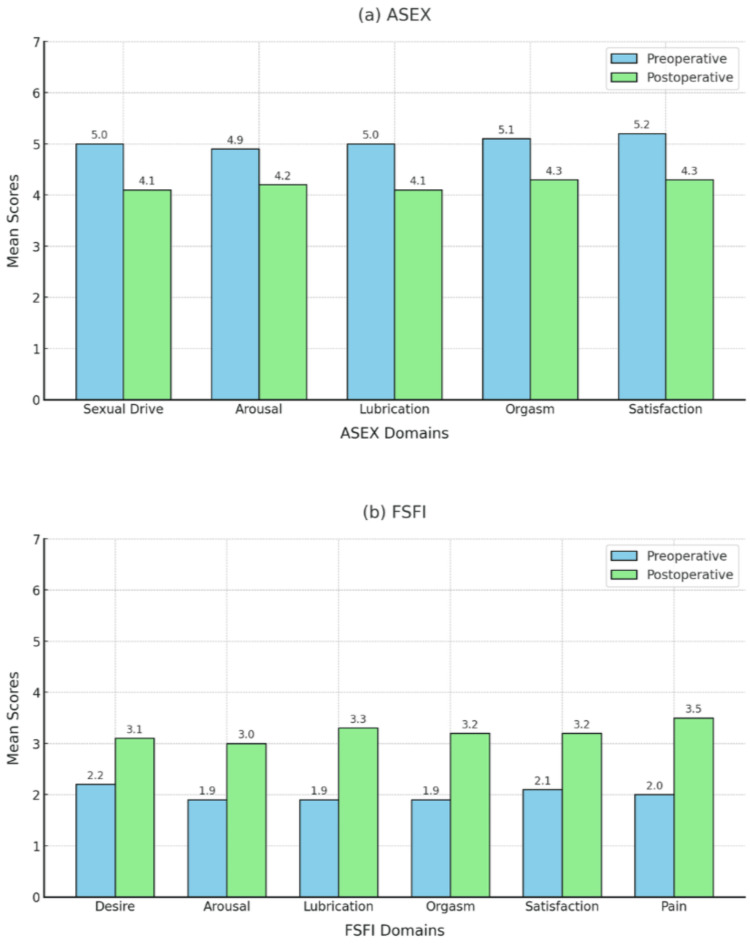
Comparison of the ASEX and FSFI mean scores from baseline to six months after surgery for the study participants (n=60). ASEX: Arizona Sexual Experiences Scale; FSFI: Female Sexual Function Index.

The mean ASEX and FSFI scores showed improvement in SF from preoperative to postoperative evaluation in both groups, as summarized in Table 3 and Figure 2. Improvements were observed in all SF domains, including sexual desire, arousal, lubrication, orgasm, satisfaction, and pain.

**Table 3:** Mean ASEX and FSFI scores from preoperative to postoperative evaluation in the TH and STH groups. TH: Total hysterectomy; STH: Subtotal hysterectomy; n: Frequencies; ASEX: Arizona Sexual Experiences Scale; FSFI: Female Sexual Function Index. Notes*:* ^a^ preoperative results; ^b^ postoperative results.

	TH (n=30)		P-value	STH (n=30)		P-value
	Pre-op^ a^	Post-op^ b^		Pre-op^ a^	Post-op^ b^	
ASEX domains, mean score±SD						
Desire	5.0±1.0	4.2±0.9	.005	4.9±0.8	4.0±0.7	< 0.001
Arousal	5.0±0.9	4.4±0.9	.024	4.8±0.6	4.0±0.8	< 0.001
Lubrication	5.0±0.9	4.3±0.8	.007	4.9±0.5	3.9±0.8	< 0.001
Orgasm	5.2±0.8	4.5±0.8	.002	5.0±0.6	4.1±0.9	< 0.001
Satisfaction	5.3±0.9	4.5±0.8	.005	5.0±0.8	4.1±1.0	0.001
FSFI domains, mean score±SD						
Desire	2.1±1.0	3.0±0.9	.004	2.3±0.9	3.1±1.0	0.003
Arousal	1.6±1.5	2.7±1.1	.001	2.1±1.4	3.2±1.0	0.001
Lubrication	1.7±1.7	2.8±1.3	.007	2.0±1.4	3.6±1.1	< 0.001
Orgasm	1.6±1.6	2.8±1.3	.003	2.1±1.5	3.5±1.1	0.001
Satisfaction	1.9±1.2	2.9±1.2	.005	2.2±1.3	3.4±1.1	0.004
Pain	1.7±1.7	3.0±1.2	.004	2.3±1.6	3.9±1.4	0.002
FSFI total score	10.7±8.7	17.5±6.7	.003	13.2±7.6	21.1±6.3	0.001

The preoperative mean ASEX score was 25.1, suggesting compromised SF, particularly in the orgasm and satisfaction domains. After hysterectomy, both groups exhibited a notable reduction in ASEX scores, with the aggregate mean score declining to 21.1, reflecting an improvement in SF post-surgery.

For the FSFI, the preoperative results showed a score of 11.9, indicative of pronounced sexual dysfunction within both the TH and STH cohorts. Although the TH group showed marginally lower FSFI scores compared to the STH group, this difference did not reach statistical significance. In particular, only 6.7% of the individuals in both cohorts achieved a preoperative FSFI score above the normative threshold of 26.55. After surgical intervention, there was a notable improvement in SF evidenced by an increase in the mean FSFI score to 19.3. Postoperative FSFI scores that exceeded the normal threshold of 26.55 were observed in 10% of the TH group and a significantly higher proportion of 26.7% in the STH group. Despite the observed improvements in SF, the statistical analysis did not reveal significant differences in the magnitude of the change in these scores before and after surgery between the TH and STH groups (Table 4).

**Table 4:** Comparison of the change in median scores of the ASEX and FSFI from baseline to six months after surgery between the two interventions TH: Total hysterectomy; STH: Subtotal hysterectomy; n: Frequencies; ASEX: Arizona Sexual Experiences Scale; FSFI: Female Sexual Function Index.

Scale	Change in medians (range)		P-value
	TH (n=30)	STH (n=30)	
ASEX domains			
Desire	-1.0 (-1.0 to 0.0)	-1.0 (-2.0 to 0.0)	0.791
Arousal	-1.0 (-1.2 to 0.0)	-1.0 (-2.0 to 0.0)	0.59
Lubrication	-1.0 (-1.0 to 0.0)	-1.0 (-2.0 to 0.0)	0.34
Orgasm	-1.0 (-1.0 to 0.0)	-1.0 (-2.0 to 0.0)	0.674
Satisfaction	-1.0 (-1.2 to 0.0)	-1.0 (-2.0 to 0.0)	0.554
FSFI domains			
Desire	1.2 (0.0-1.8)	1.2 (0.0-1.8)	0.922
Arousal	1.3 (0.6-2.4)	1.6 (0.1-2.1)	0.881
Lubrication	1.5 (0.8-1.8)	1.8 (1.2-3.0)	0.249
Orgasm	1.4 (0.6-2.1)	2.0 (-0.1 to 2.7)	0.521
Satisfaction	1.2 (0.4-2.4)	1.6 (-0.9 to 2.4)	0.667
Pain	1.6 (0.6-3.2)	1.8 (-0.6 to 3.8)	0.772
Total score	9.1 (4.3-11.5)	9.8 (-0.5 to 15.8)	0.544

Furthermore, the duration until the resumption of sexual activity after surgery did not differ significantly between surgical types, with both TH and STH groups experiencing a median delay of 50 and 47.5 days, respectively. Furthermore, comparative analysis of SF between the two surgical approaches, laparotomy versus the vaginal route, within the TH cohort did not produce statistically significant differences.

## Discussion

Our analysis did not reveal statistically significant differences in SF scores (ASEX and FSFI) before and after the operation between the TH and STH groups, with p-values greater than 0.05. This lack of significant difference extends to the timeline for resuming sexual activity after surgery. The postoperative evaluation for both cohorts revealed a notable improvement in SF, which was quantitatively supported by the ASEX and FSFI results.

The impact of hysterectomy on sexuality has been a subject of interest for many researchers, as it is a significant concern for both patients and surgeons. The published literature presents conflicting results. A substantial review of the literature, comprising 34 studies [16], predominantly indicated an improvement in SF after hysterectomy. This was particularly evident in patients who were sexually active before the operation, with many reporting sustained or enhanced sexual performance postoperatively. The predominant explanation for our findings lies in the relief of preoperative symptoms, which correlates with a return to normal sexual activity and increased sexual satisfaction [17]. This observation is in alignment with the findings of the Maine Women's Health Study, wherein Carlson KJ et al. [18] demonstrated the significant efficacy of hysterectomy in mitigating symptoms related to common benign gynecological conditions, resulting in a notable improvement in SF. The study underlines the proposition that the improvement in SF after hysterectomy is primarily due to the relief of symptoms facilitated by surgical intervention [18]. Further supporting this argument, our study does not discern appreciable differences attributable to the surgical method used, indicating that the benefits in SF post-hysterectomy are fundamentally linked to symptom relief rather than the specifics of the surgical approach. While symptom relief undoubtedly contributes to improved SF after hysterectomy, as supported by our findings, it is imperative to also consider psychological factors, the quality of partner relationships, and patient expectations before and after surgery.

On the other hand, a systematic review and meta-analysis [11] found that hysterectomy was not associated with significant changes in female SF. However, Dedden SJ et al. [11] stated that the studied population was too heterogeneous to determine the direction of changes in SF. The conclusion drawn from the meta-analysis was tempered by the recognition that the absence of evidence is not synonymous with evidence of absence. Furthermore, Lonnée-Hoffmann R and Pinas I [19] explored the potential negative impact of hysterectomy on SF, highlighting the risk of long-term health problems and sexual dysfunctions such as diminished sexual pleasure, frequency, and comfort. These dysfunctions were attributed to hormonal imbalances, specifically reductions in androgen and estrogen, resulting from the surgery.

A possible explanation for the observed heterogeneity in results across the literature may be attributed not to the hysterectomy procedure itself but rather to the underlying surgical indications, which can predict the degree of alteration in SF after hysterectomy. It is possible that individuals who undergo hysterectomy for conditions such as fibroids or menorrhagia may report improvements in SF due to the resolution of these specific complaints. On the contrary, individuals who undergo hysterectomy for chronic pelvic pain might continue to experience postoperative pain, resulting in negligible improvement in SF. Consequently, future investigations should focus on analyzing changes in SF after hysterectomy within homogeneous cohorts delineated by surgical indications.

In evaluating the impact of the surgical approach on SF comparing TH with STH, our findings did not indicate significant superiority of one method over the other. This outcome is consistent with the prevailing body of literature. A recent systematic review and meta-analysis [11], along with previous clinical trials [8,20-22], have suggested that TH does not demonstrate inferiority to STH in terms of SF variations observed from baseline to the postoperative phase. In addition, a comprehensive Cochrane review in 2012 [23] incorporated data from six randomized controlled trials (RCTs) conducted between 2002 and 2010, all of which reported on SF outcomes with follow-up periods extending up to two years. A meta-analysis [23] revealed no statistically significant differences in terms of sexual satisfaction or patient-reported dyspareunia between subtotal and TH. Further emphasizing the robustness of these findings, one RCT [24] included in the Cochrane review, which extended its follow-up to five years, similarly reported no differences in sexual satisfaction between the two hysterectomy techniques.

It should be noted that any major pelvic surgery or injury carries the potential risk of damaging nerves and blood vessels essential for normal SF. Surgeons currently lack precise knowledge about the location of these vital anatomical structures in the female pelvis, highlighting the need for further research to prevent unintentional damage during surgery. By providing comprehensive information about the sexual effects of hysterectomies, healthcare providers can support patients in making informed decisions and addressing their concerns. Ultimately, optimizing patient satisfaction and overall quality of life should remain a key objective in the management of benign gynecological conditions requiring hysterectomy.

Limitations

Our study has several limitations that warrant consideration. First and foremost, the modest cohort size limits the statistical power to detect minor but potentially clinically relevant differences. This raises the question of the clinical importance of potential differences that remain undetected in a sample of 60 patients. Furthermore, the absence of randomization in the allocation of patients to the specific type of hysterectomy performed introduces the possibility of confounding by baseline disparities in factors affecting sexual well-being. An ideal research design would have been a RCT; however, the recruitment of a sufficient number of gynecologists for such a study proved challenging, limiting our ability to conduct it. Another constraint is the participation of multiple surgeons in the procedures, all within a single center setting. This aspect may detract from the external validity of our findings, as the results may not be generalizable across different surgical environments or physician expertise. Future research could benefit from a large multicenter prospective cohort study to improve the generalizability of the findings. Lastly, we acknowledge that the follow-up period did not allow an evaluation of longer-term outcomes of hysterectomy, such as the potential for early menopause onset and tissue-related complications, including pelvic prolapse and incontinence, which can manifest within 15 years after surgery [25]. Addressing these long-term effects in future research is crucial for a more comprehensive understanding of the impact of hysterectomy on sexual and overall health.

## Conclusions

The findings of our study indicated that hysterectomy had a positive impact on overall SF, which might be explained by symptomatic relief from conditions that warrant hysterectomy. Furthermore, there were no statistically significant differences in SF between TH and STH. Therefore, preservation of the cervix with the aim of improving overall sexual satisfaction cannot be recommended. Women who require hysterectomy should be informed about the results of previous studies mentioned earlier, enabling them to make informed decisions about the most suitable procedure.

## References

[1] Hammer A, Rositch AF, Kahlert J, Gravitt PE, Blaakaer J, Søgaard M (2015). Global epidemiology of hysterectomy: possible impact on gynecological cancer rates. Am J Obstet Gynecol.

[2] Ramdhan RC, Loukas M, Tubbs RS (2017). Anatomical complications of hysterectomy: a review. Clin Anat.

[3] Chevrot A, Margueritte F, Fritel X, Serfaty A, Huchon C, Fauconnier A (2021). Hysterectomy: practices evolution between 2009 and 2019 in France. Gynecol Obstet Fertil Senol.

[4] Monterrosa-Castro A, Monterrosa-Blanco A, Beltrán-Barrios T (2018). Insomnia and sexual dysfunction associated with severe worsening of the quality of life in sexually active hysterectomized women. Sleep Sci.

[5] Lopès P, Poudat F-X (2022). Manuel de sexologie. Manuel de sexologie. 4th ed.

[6] Komisaruk BR, Whipple B (2005). Functional MRI of the brain during orgasm in women. Annu Rev Sex Res.

[7] Cour F, Droupy S, Faix A, Methorst C, Giuliano F (2013). Anatomy and physiology of sexuality. Prog Urol.

[8] Kuppermann M, Summitt RL Jr, Varner RE (2005). Sexual functioning after total compared with supracervical hysterectomy: a randomized trial. Obstet Gynecol.

[9] federici francesca, Greta B, Teresa P (2020). Total versus subtotal hysterectomy for benign uterine disease: which advantages. Res Square.

[10] Saccardi C, Gizzo S, Noventa M (2015). Subtotal versus total laparoscopic hysterectomy: could women sexual function recovery overcome the surgical outcomes in pre-operatory decision making?. Arch Gynecol Obstet.

[11] Dedden SJ, Werner MA, Steinweg J, Lissenberg-Witte BI, Huirne JA, Geomini PM, Maas JW (2023). Hysterectomy and sexual function: a systematic review and meta-analysis. J Sex Med.

[12] Briki M, Haffen E, Monnin J, Tio G, Nicolier M, Sechter D, Vandel P (2014). Sexual dysfunction and depression: validity of a French version of the ASEX scale. Encephale.

[13] Zakhour S, Sardinha A, Levitan M, Berger W, Nardi AE (2022). Instruments for assessing sexual dysfunction in Arabic: a systematic literature review. Transcult Psychiatry.

[14] Hevesi K, Mészáros V, Kövi Z, Márki G, Szabó M (2017). Different characteristics of the female sexual function index in a sample of sexually active and inactive women. J Sex Med.

[15] Wylomanski S, Bouquin R, Philippe HJ (2014). Psychometric properties of the French Female Sexual Function Index (FSFI). Qual Life Res.

[16] Danesh M, Hamzehgardeshi Z, Moosazadeh M, Shabani-Asrami F (2015). The effect of hysterectomy on women's sexual function: a narrative review. Med Arch.

[17] Kazemi F, Alimoradi Z, Tavakolian S (2022). Effect of hysterectomy due to benign diseases on female sexual function: a systematic review and meta-analysis. J Minim Invasive Gynecol.

[18] Carlson KJ, Miller BA, Fowler FJ Jr (1994). The Maine Women's Health Study: I. Outcomes of hysterectomy. Obstet Gynecol.

[19] Lonnée-Hoffmann R, Pinas I (2014). Effects of hysterectomy on sexual function. Curr Sex Health Rep.

[20] Learman LA, Summitt RL, Varner RE (2003). A randomized comparison of total or supracervical hysterectomy: surgical complications and clinical outcomes. Obstet Gynecol.

[21] Thakar R, Ayers S, Clarkson P, Stanton S, Manyonda I (2002). Outcomes after total versus subtotal abdominal hysterectomy. N Engl J Med.

[22] Ellström Engh MA, Jerhamre K, Junskog K (2010). A randomized trial comparing changes in sexual health and psychological well-being after subtotal and total hysterectomies. Acta Obstet Gynecol Scand.

[23] Lethaby A, Mukhopadhyay A, Naik R (2012). Total versus subtotal hysterectomy for benign gynaecological conditions. Cochrane Database Syst Rev.

[24] Andersen LL, Zobbe V, Ottesen B, Gluud C, Tabor A, Gimbel H (2015). Five-year follow up of a randomised controlled trial comparing subtotal with total abdominal hysterectomy. BJOG.

[25] Madueke-Laveaux OS, Elsharoud A, Al-Hendy A (2021). What we know about the long-term risks of hysterectomy for benign indication-a systematic review. J Clin Med.

